# IMGG: Integrating Multiple Single-Cell Datasets through Connected Graphs and Generative Adversarial Networks

**DOI:** 10.3390/ijms23042082

**Published:** 2022-02-14

**Authors:** Xun Wang, Chaogang Zhang, Ying Zhang, Xiangyu Meng, Zhiyuan Zhang, Xin Shi, Tao Song

**Affiliations:** 1 College of Computer Science and Technology, China University of Petroleum, Qingdao 266555, China; wangsyun@upc.edu.cn (X.W.); s20070030@s.upc.edu.cn (C.Z.); zhangy9808@163.com (Y.Z.); xiangyumeng@s.upc.edu.cn (X.M.); flyeagle237@163.com (Z.Z.); shix1104@163.com (X.S.); 2 Department of Artificial Intelligence, Faculty of Computer Science, Campus de Montegancedo, Polytechnical University of Madrid, Boadilla del Monte, 28660 Madrid, Spain

**Keywords:** scRNA-seq, batch effect, connected graphs, deep learning, GAN

## Abstract

There is a strong need to eliminate batch-specific differences when integrating single-cell RNA-sequencing (scRNA-seq) datasets generated under different experimental conditions for downstream task analysis. Existing batch correction methods usually transform different batches of cells into one preselected “anchor” batch or a low-dimensional embedding space, and cannot take full advantage of useful information from multiple sources. We present a novel framework, called IMGG, i.e., integrating multiple single-cell datasets through connected graphs and generative adversarial networks (GAN) to eliminate nonbiological differences between different batches. Compared with current methods, IMGG shows excellent performance on a variety of evaluation metrics, and the IMGG-corrected gene expression data incorporate features from multiple batches, allowing for downstream tasks such as differential gene expression analysis.

## 1. Introduction

The maturation of single-cell RNA-sequencing (scRNA-seq) technologies and the continuing decrease in sequencing costs have encouraged the establishment of large-scale projects such as the Human Cell Atlas, which generates transcriptomic data from thousands to millions of cells and almost inevitably involves multiple batches across time points, sequencing technologies, or experimental protocols [1,2]. With the explosive accumulation of single-cell studies, integrative analysis of data from experiments of different contexts is particularly important. However, batch effects or systematic differences in gene expression profiles across batches not only can obscure the true underlying biology but also may lead to spurious findings [3,4,5]. Therefore, to avoid misleading conclusions, they must be corrected before further analysis.

In recent years, a number of algorithms have been published for batch-effect correction. There are two main categories of these methods, according to the correction results. The first is to select a batch as “anchor” and convert other batches to the “anchor” batch, e.g., MNN [6], iMAP [7], SCALEX [8], etc., which has the advantage that different batches of cells can be converted to one other so that gene expression can be studied under the same experimental conditions, and the disadvantage that it is not possible to fully combine the features of each batch and it is difficult to select an “anchor” batch because the cell types contained in each batch are unknown. The other is to transform all batches of data into a low-dimensional space to correct batch effects, e.g., Scanorama [9], Harmony [10], DESC [11], BBKNN [12], etc., which has the advantage of extracting biologically relevant latent features and reducing the impact of noise, and the disadvantage that it cannot be used for differential gene expression analysis.

To compensate for the shortcomings of these methods, we integrate multiple single-cell datasets through connected graphs and generative adversarial networks (GAN) to design a batch-effect correction framework called IMGG. IMGG first finds mutual nearest neighbor pairs (MNNs) multiple times in the low-dimensional embedding space, then constructs cross-batch similar-cell connected graphs by MNNs and builds an intermediate batch based on the similarity relationship of cells on these graphs, finally correcting the batch effects by transforming all batches of cells to the intermediate batch with GAN. Our experiments on multiple datasets demonstrate that IMGG is superior in various evaluation metrics compared to other algorithms; moreover, the IMGG-corrected data can improve gene differential expression analysis. Finally, according to different task goals, we give some recommendations for the use of batch-correction methods.

## 2. Results

**Figure 1:**
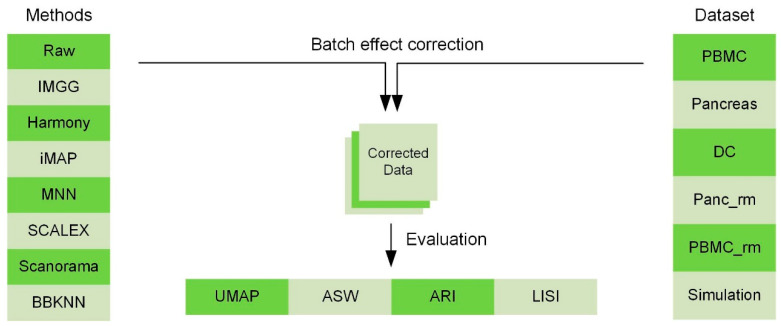
Comparing the performance of IMGG with other algorithms on multiple datasets using four evaluation metrics.

To show the superiority of IMGG, we designed multiple datasets and used four evaluation methods to compare its ability to mix batches while maintaining cell-type separation with current prevalent algorithms (Figure 1). The datasets covered two batches, *n* (*n* > 2) batches, non-overlapping, and simulation data, respectively (Appendix A.2). All comparison methods, which have attracted a lot of attention from researchers in recent years, included MNN and its variants, which corrected batch effects by transforming all batches to a particular batch or embedding them into a low-dimensional space (Appendix A.3). To evaluate the batch-correction effect, we used the Uniform Manifold Approximation and Projection (UMAP) [13] visualizations, average silhouette width (ASW) [14], adjusted rand index (ARI) [15], and local inverse Simpson’s index(LISI) [10] benchmarking metrics (Appendix A.1). UMAP plots visualized the changes in different batch data before and after correcting batch effects, and ASW, ARI, and LISI metrics were used to assess the ability to mix batches and maintain cell-type separation [16]. For easy comparison, ASW scores were plotted as 1 − ASW batch and ASW cell type, and ARI scores were calculated and plotted in the same manner (1 − ARI batch and ARI cell type). For the LISI metric, we calculated the mean of all cell scores and plotted the scores as 1 − cLISI and iLISI, so that a higher value would indicate better performance. All evaluation methods were calculated for common cell types only, and to summarize these metrics, we summed the scores of IMGG and other algorithms according to their ranking on each evaluation method, so that a lower sum of ranking scores indicates better overall algorithm performance. Specific experimental results are presented below.

### 2.1. IMGG Outperforms Existing Methods on Two Batches of Overlapping Data

We first showed the performance of IMGG in correcting two batches of overlapping data using the human peripheral blood mononuclear cell(PBMC) dataset [16,17], which comprised ‘pbmc_3p’ batch obtained by 10× 3’ Genomics protocols and ‘pbmc_5p’ batch obtained by 10× 5’ Genomics protocols.

**Figure 2:**
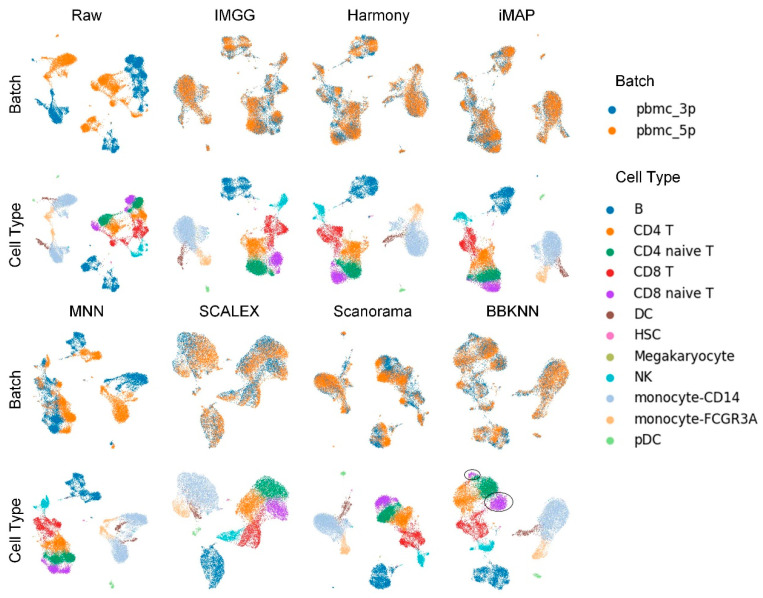
Qualitative evaluation of 7 batch-effect correction methods using UMAP visualization for PBMC dataset. The UMAP diagrams of raw data, IMGG, Harmony, and iMAP are plotted in the top half, and the UMAP diagrams of MNN, SCALEX, Scanorama, and BBKNN are plotted in the bottom half. Each half contains two rows of UMAP plots. In the first row, cells are colored by batch, and in the second by cell type.

The UMAP visualization plots (Figure 2) showed a large deviation between the two batches of cells in the raw data after preprocessing. Except for the MNN method, IMGG and all other methods could successfully mix the common cells; the “kissing effects” (where the different types of cells are not clearly separated on the visualization plot and their borders are close together) was obvious in SCALEX; the ‘CD8 naive T’ cells were separated into two parts in BBKNN; and IMGG, Harmony, and iMAP, as well as Scanorama could achieve good results in differentiating cell types.

**Figure 3:**
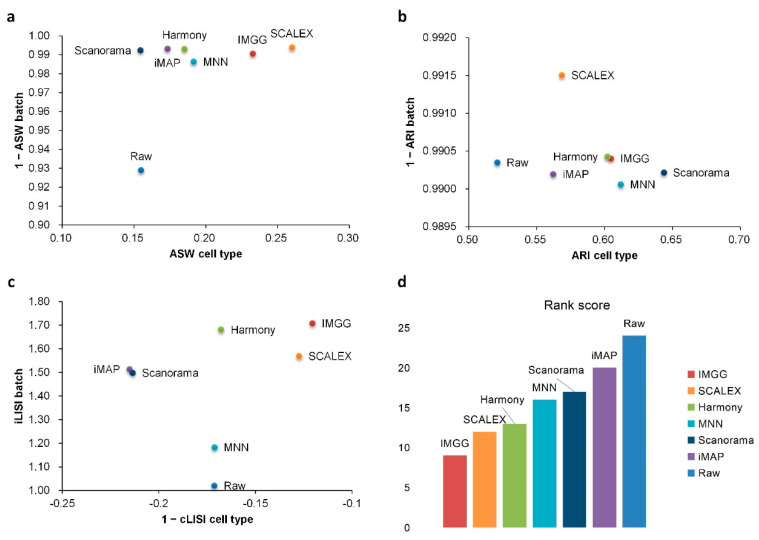
Quantitative evaluation of 6 batch-effect correction methods using the three-assessment metrics (**a**) ASW, (**b**) ARI, and (**c**) LISI on PBMC dataset. Methods appearing at the upper-right quadrant of the ASW, ARI, and LISI plots are the good performing methods. (**d**) The sum of each method’s ranking on all evaluation metrics, with smaller values indicating better overall performance. Detailed data are recorded in Table A5.

For ASW (Figure 3a), both IMGG and other methods obtained good scores in batch mixing (1 − ASW batch > 0.98), and in cell-type purity score IMGG was second only to SCALEX. For ARI (Figure 3b), both IMGG and other methods obtained good scores (1 − ARI batch > 0.99), and in cell-type purity IMGG scores ranked third. For LISI (Figure 3c), IMGG ranked highest in both cell-type purity metric cLISI and batch-mixing metric iLISI. Finally, based on the sum of the rankings of the evaluated metrics (for fairness, if the score difference was less than 0.01, the ranking was considered the same), IMGG ranked first (Figure 3d).

### 2.2. IMGG Outperforms Existing Methods on Multiple Batches of Overlapping Data

To show the advantage of IMGG in processing multiple batches, we compared its performance with the current mainstream algorithms using the human pancreas (Pancreas) dataset [18,19,20,21,22], which contained five batches of data obtained by different techniques.

**Figure 4:**
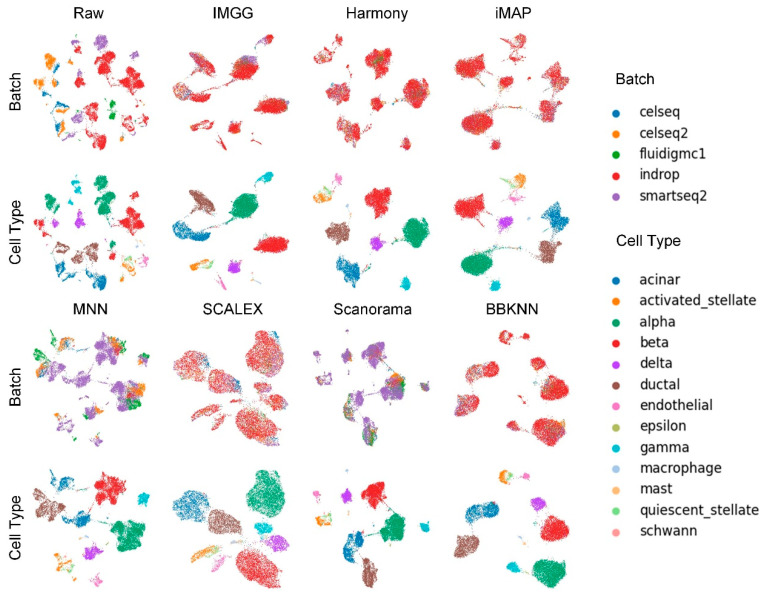
Qualitative evaluation of 7 batch-effect correction methods using UMAP visualization for Pancreas dataset. The UMAP diagrams of raw data, IMGG, Harmony, and iMAP are plotted in the top half, and the UMAP diagrams of MNN, SCALEX, Scanorama, and BBKNN are plotted in the bottom half. Each half contains two rows of UMAP plots. In the first row, cells are colored by batch, and in the second by cell type.

The UMAP visualization plots (Figure 4) showed that the preprocessed raw data had large batch effects, the MNN algorithm could only pull together different batches and could not mix batches well, while IMGG and the other five methods all mixed different batches and distinguished different cell types well.

**Figure 5:**
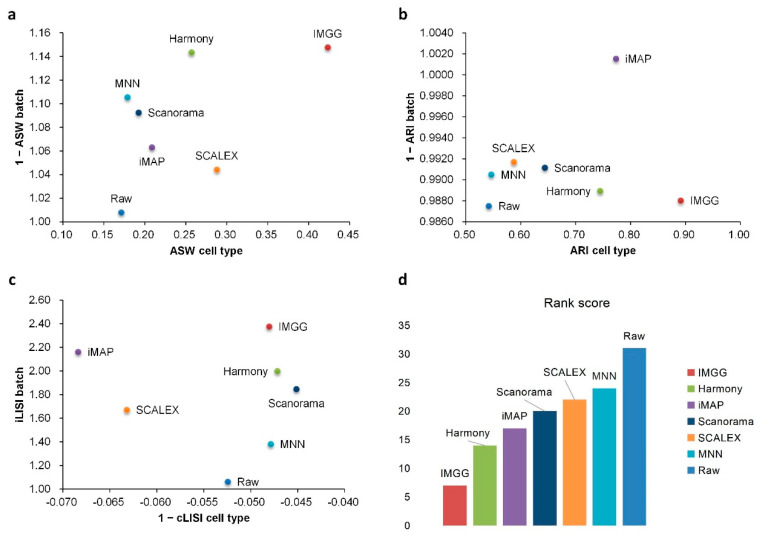
Quantitative evaluation of 6 batch-effect correction methods using the three-assessment metrics (**a**) ASW, (**b**) ARI, and (**c**) LISI on Pancreas dataset. Methods appearing at the upper-right quadrant of the ASW, ARI, and LISI plots are the good performing methods. (**d**) The sum of each method’s ranking on all evaluation metrics, with smaller values indicating better overall performance. Detailed data are recorded in Table A6.

For ASW (Figure 5a), IMGG was ahead of other methods in both batch-mix score and cell-purity score. For ARI (Figure 5b), all methods performed well in batch-mix score (1 − ARI batch > 0.98), and IMGG was ahead of other methods in cell-type purity. For LISI (Figure 5c), IMGG had the highest score in batch-mix index iLISI; and in the cell-type purity index cLISI, IMGG, Harmony, MNN, and Scanorama were comparable (score difference less than 0.01). Finally, based on the sum of the rankings of the evaluated indicators (for fairness, if the score difference was less than 0.01, the ranking was considered the same), IMGG ranked first (Figure 5d).

### 2.3. IMGG Outperforms Existing Methods on Non-Overlapping Data

In practical studies of scRNA-seq, cell types usually differ between batches. Therefore, we again performed experiments on non-overlapping data to demonstrate the ability of IMGG to handle real data.

Human dendritic cells (DC) were a two-batch dataset obtained using Smart-seq2 technology, and consisted of four types of human dendritic cells (DCs), i.e., CD1C DC, CD141 DC, plasmacytoid DC (pDC), and double-negative cells (DoubleNeg) [23]. Two types of biologically similar cells, CD1C DC from batch1 and CD141 DC from batch2, were removed to ensure the two sub-datasets contained batch-specific cells [16].

We first conducted experiments using the DC dataset to demonstrate the ability of IMGG in handling two batches of non-overlapping data.

**Figure 6:**
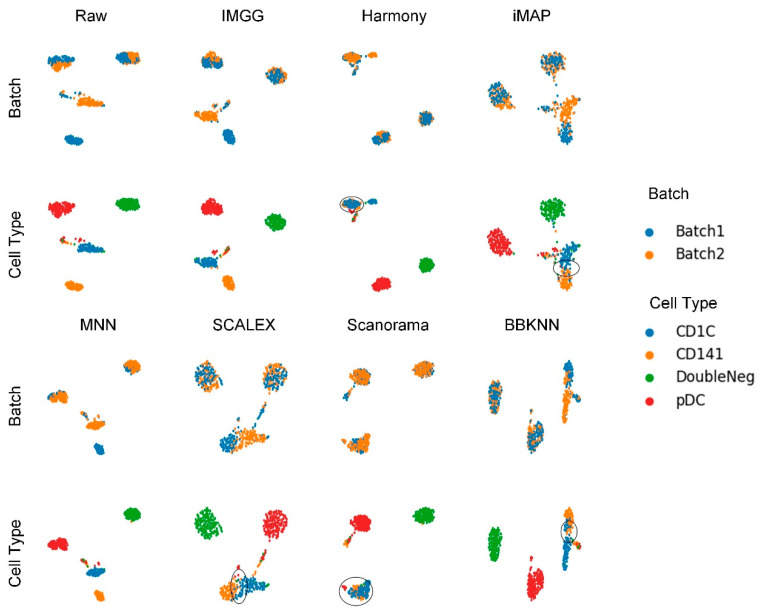
Qualitative evaluation of 7 batch-effect correction methods using UMAP visualization for DC dataset. The UMAP diagrams of raw data, IMGG, Harmony and iMAP are plotted in the top half, and the UMAP diagrams of MNN, SCALEX, Scanorama, and BBKNN are plotted in the bottom half. Each half contains two rows of UMAP plots. In the first row, cells are colored by batch, and in the second by cell type.

The UMAP visualization plots (Figure 6) showed only a “kissing effect” between the two batches in the preprocessed raw data, indicating small batch effects. After running batch-correction algorithms, the two batch-specific cell types ‘CD1C’ and ‘CD141’ overlapped incorrectly in Harmony and Scanorama, and there was still a “kiss effect” on iMAP and SCALEX, as well as BBKNN. Only IMGG and MNN can correctly distinguish different cell types.

**Figure 7:**
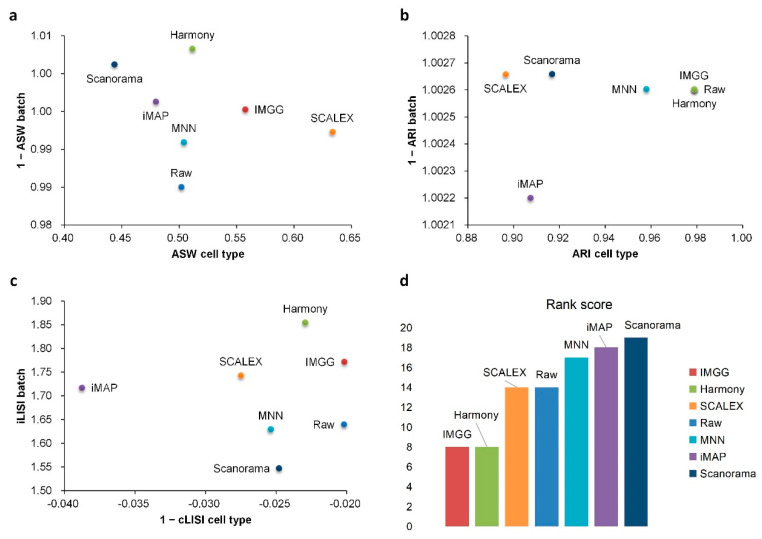
Quantitative evaluation of 6 batch-effect correction methods using the three-assessment metrics (**a**) ASW, (**b**) ARI, and (**c**) LISI on DC dataset. Methods appearing at the upper-right quadrant of the ASW, ARI, and LISI plots are the good performing methods. (**d**) The sum of each method’s ranking on all evaluation metrics, with smaller values indicating better overall performance. Detailed data are recorded in Table A7.

For ASW (Figure 7a), all methods performed well in mixing batches (1 − ASW > 0.99) and IMGG was next to SCALEX in cell-type purity assessment. For ARI (Figure 7b), all methods performed well in batch mixing (1 − ARI batch > 1), and IMGG was tied with Harmony for first place in cell-type purity. For LISI (Figure 7c), IMGG was second only to Harmony in batch-mixing assessment, and ranked first in cell-type purity assessment. Finally, based on the sum of the rankings of the assessment metrics (for fairness, if the score difference was less than 0.01, the ranking was considered the same), IMGG ranked first alongside Harmony (Figure 7d), but the UMAP visualization plots suggested that Harmony was not well suited to handle this type of data.

We also demonstrated the ability of IMGG to handle multiple batches of non-overlapping data (Appendix A.5).

### 2.4. IMGG-Corrected Data Can Integrate Features from Multiple Batches

The novelty of IMGG is the ability to combine the features of different batches. We first performed differential expression analysis using B cells from the PBMC dataset between the ‘pbmc_3p’ batch and the ‘pbmc_5p’ batch to filter out the significant genes causing the separation of the two batches of B cells. The IMGG batch-correction algorithm was then run by selecting the Mean, Max, and Min modes, respectively, and the corrected data were subjected to differential expression analysis again, and no genes were screened out, which demonstrated that each pattern could eliminate the differences between the two batches.

**Figure 8:**
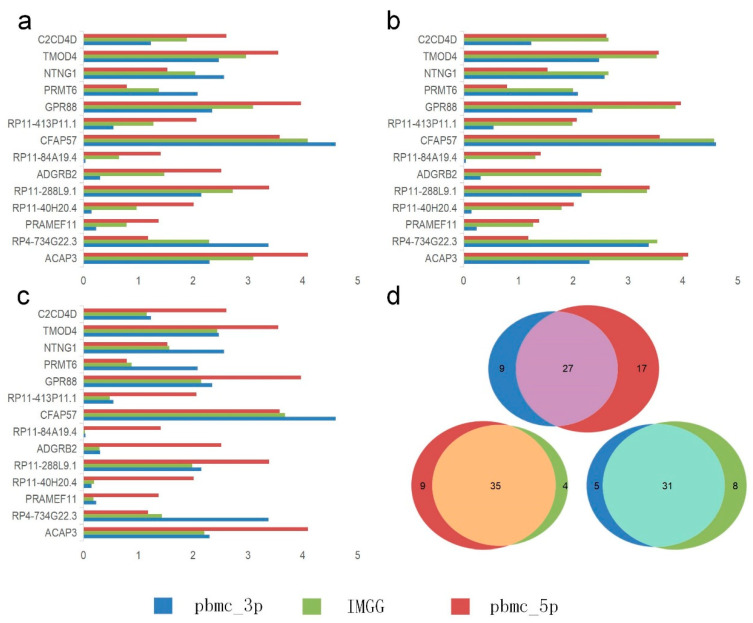
Gene differential expression analysis. (**a**–**c**) Differential expression of significant genes on B cells in the PMBC dataset before and after IMGG correction, where (**a**) Mean pattern, (**b**) Max pattern, and (**c**) Min pattern. (**d**) Venn diagram of changes in the number of differentially expressed genes of B cells and CD4 T cells in the PBMC dataset before and after IMGG correction. The original data with ‘pbmc_3p’ batch was colored in blue and ‘pbmc_5p’ in red. The IMGG-corrected data no longer distinguished between batches and used lime-green coloring.

We visualized the changes in expression of significant genes causing B-cell segregation before and after correction. In the Mean pattern (Figure 8a) the expression of significant genes changed toward their means (i.e., for a single gene, the expression after IMGG correction was approximately equal to the mean of the expression in the two batches before correction). In the Max pattern (Figure 8b) the expression of significant genes changed toward their maxima. In the Min pattern (Figure 8c) the expression of significant genes changed toward their minima.

**Figure A4:**
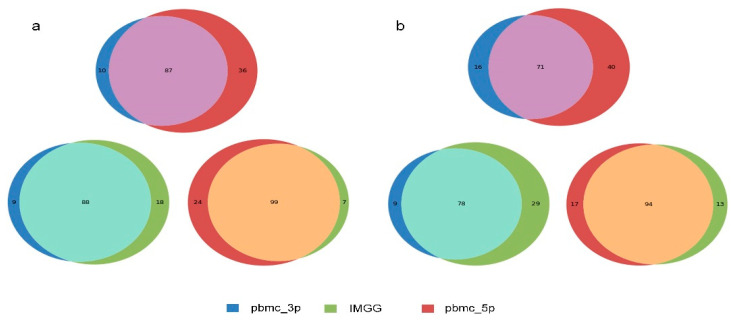
(**a**) Venn diagram of changes in the number of differentially expressed genes of NK cells and DC cells in the PBMC dataset before and after IMGG correction. (**b**) Venn diagram of changes in the number of differentially expressed genes of CD8 T cells and monocyte-CD14 cells in the PBMC dataset before and after IMGG correction. The original data with ‘pbmc_3p’ batch was colored in blue and ‘pbmc_5p’ in red. The IMGG-corrected data no longer distinguished between batches and used lime green coloring.

Meanwhile, we showed IMGG’s ability in finding differentially expressed genes by combining multi-batch features. We performed differential expression analysis using B cells and CD4 T cells from PBMC dataset in ‘pbmc_3p’ batch, ‘pbmc_5p’ batch, and IMGG corrected data, and the number of filtered differentially expressed genes was visualized by Venn diagram, respectively (Figure 8d; similarly, the Venn diagrams of NK cells and DC cells, as well as CD8 T cells and monocyte-CD14 cells are shown in Figure A4). As can be seen from the figure, the differentially expressed genes found in the ‘pbmc_3p’ batch and the ‘pbmc_5p’ batch are more different, but the differentially expressed genes found after IMGG correction are more similar to the genes found in each batch individually, which indicated that the differential expression analysis using the IMGG-corrected data can filter genes that incorporate both batches’ characteristics and better reflect the true differences. To prove the above conclusion, we used the expression of these three sets of differentially expressed genes on raw data, ‘pbmc_3p’ batch, ‘pbmc_5p’ batch, and IMGG-corrected data for ASW assessment of the two cell types, respectively, and higher ASW scores indicated that the two cell types were more dissimilar, and the experimental results showed that the differentially expressed genes found using IMGG-corrected data achieved the best ASW scores (Appendix A.6), which confirmed that the IMGG can improve differential expression analysis.

### 2.5. IMGG Performs at an Excellent Level in Terms of Time Overhead

To test the time-performance of IMGG, we simulated datasets of 500–100,000 cells and compared the runtime of IMGG and other methods on these datasets, respectively.

**Figure 9:**
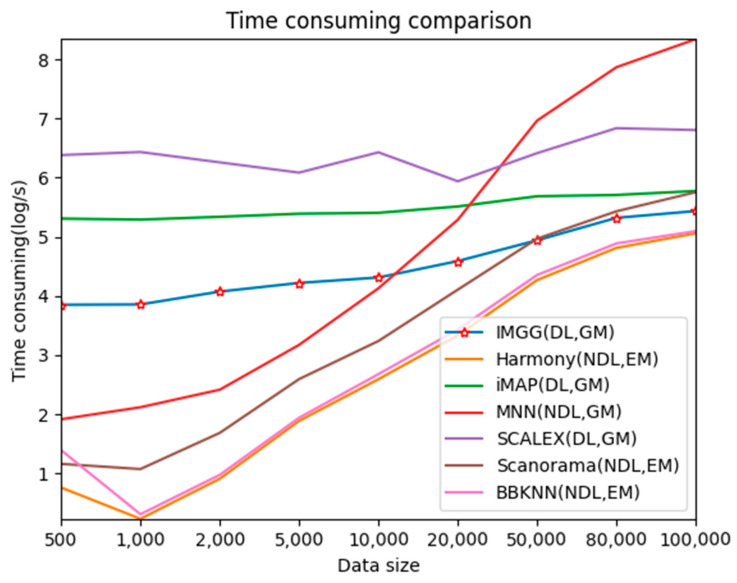
Time-consuming comparison between IMGG and other methods on different size datasets. DL means deep learning is used and NDL means no deep learning is used. GM means corrected gene expression matrix is returned and EM means corrected embedding matrix is returned.

For better presentation, we logarithmized the running time (Figure 9). The three deep learning-based methods, IMGG, iMAP, and SCALEX, all have a larger time overhead than the other non-deep learning methods on small datasets because there is an additional training process using deep learning techniques. The time spent by these three methods increases at a lower rate than the other methods as the data size increases, and the time complexity approximates O(log), and IMGG outperforms iMAP and SCALEX. The running time of the methods that return corrected gene expression matrices is larger than that of the methods that return reduced dimensional matrices, but this gap decreases as the data size increases. In summary, the time complexity of IMGG is better than that of the same class method.

## 3. Discussion

IMGG provides a solution to the batch effects present in two-batch, multi-batch, and non-overlapping single-cell RNA-seq datasets. It takes the gene expression profile matrices from different batches as inputs, and outputs the corrected expression profiles. Our model combines connected graphs and generative adversarial networks, first breaking the convention that the MNN algorithm is performed only once by finding MNNs multiple times in PCA low-dimensional space, and then using MNNs to construct cross-batch similar cell connected graphs to obtain similarity relationships for all paired cells. We use the similarity relationship to build an intermediate batch as the target domain and other cells in the similar cohort as the source domain, and use GAN to perfectly mix the distributions of the shared cell types.

A remarkable feature of IMGG is that it can fully utilize the useful sides of each of the sources. We designed three patterns—Mean, Max, and Min, and demonstrated that the gene expression after IMGG correction can combine the characteristics of each batch and adjust the gene expression according to the set pattern, which may provide new insights to study the gene expression of different batches of cells.

Based on the experimental results, we give suggestions for use in different cases. If you want to obtain a low-dimensional embedding representation of gene expression, we recommend using Harmony, although IMGG uses generative adversarial networks, its performance in dimensionality reduction is not proven; if you want to obtain a graph representation of all cells, we recommend using BBKNN, although IMGG also constructs connected graphs, it does not cover all cells to improve running speed; if you want to obtain a gene expression matrix that can be used for downstream analysis, then our IMGG is recommended and it may bring you new discoveries.

In summary, extensive real-dataset benchmarking suggests that IMGG not only better rescues biological features and provides improved clustering results, but also helps to identify biologically relevant DEGs. Therefore, we anticipate that IMGG is valuable for the comprehensive analysis of multiple scRNA-seq datasets, accelerating studies involving single-cell transcriptomic gene expression.

## 4. Materials and Methods

GAN has been shown to outperform AutoEncoder-based methods in image-style migration tasks [24]. Different batches of cells are similar to different styles of images, so GAN can also be introduced to address the batch effects.

**Figure 10:**
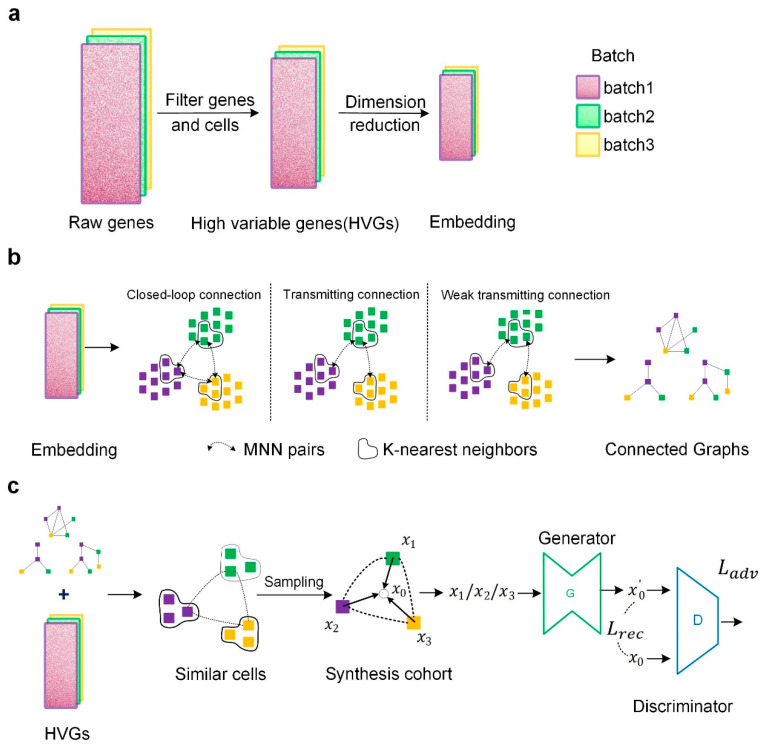
Overview of the IMGG framework. (**a**) Data preprocessing. (**b**) In the embedding space, three methods are used to construct connected graphs. (**c**) Combining the connected graphs and HVGs, sampling from similar cells, and using GAN to correct batch effects.

Our IMGG framework consists of three stages: in the first stage (Figure 10a), all genes were first preprocessed to filter out highly variable genes (HVGs), followed by transforming the HVGs to the low-dimensional embedding space by principal component analysis (PCA). In the second stage (Figure 10b), the MNN algorithm was executed multiple times to find as many different MNNs as possible between batches in the embedding space, and then MNNs were used to construct cross-batch similar-cell connected graphs. In the last stage (Figure 10c), the connected graphs obtained in the embedding space were first mapped to the HVGs’ space, and in the HVGs’ space, different batches of cells were sampled from each group of similar cells to form a synthesis queue. Then, using the middle point of the cohort synthesis as the target domain and the other cells in the cohort as the source domain, a network was trained using GAN to transform from the source domain to the target domain, and finally, the batch effect could be corrected using the trained generator. Details are further explained below.

### 4.1. Data Preprocessing

All preprocessing of the scRNA-seq datasets in this study was performed using the Scanpy package in the Python language environment [25]. 

Firstly, genes starting with ‘ERCC’, ‘MT-’, and ‘mt-’ were filtered out to prevent interference from the size of the library or the large proportion of mitochondrial gene counts. Secondly, the “scanpy.pp.filter_cells” function of Scanpy and the “ scanpy.pp.filter_genes” function exclude cells expressing fewer than 600 genes and genes expressed in fewer than 3 cells, followed by “scanpy.pp.highly_variable_genes” to select 2000 highly variable genes and normalize the data using “scanpy.pp.normalize_total”. Finally, the data were logarithmically transformed using the “scanpy.pp.log1p” function and the “scanpy.tl.pca” function was used to obtain an embedding representation of the data.

### 4.2. Constructing Cross-Batch Similar-Cell Connected Graphs

In this stage, we further explored the potential of the MNN algorithm.

First, in the low-dimensional embedding space, we executed the MNN algorithm multiple times to find MNNs (Each time, the paired cells were removed and no more than 3000 cells were sampled per batch, which could increase the diversity of paired cells and save time).

Second, to discover similar relationships between cells in different batches, we constructed connected graphs of similar cells across batches using MNNs. The construction method was consistent for overlapping and non-overlapping datasets. Here, for convenience, the construction methods are explained in terms of cells of type A appearing in three batches simultaneously.

**Figure 11:**
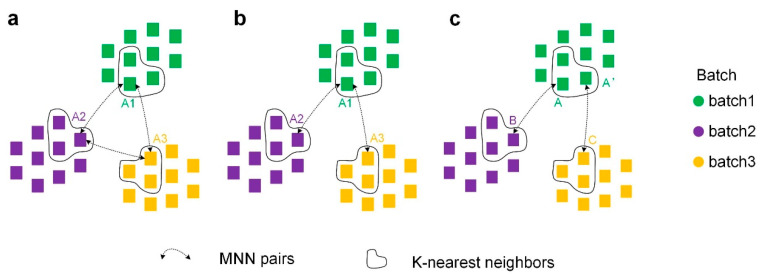
Three ways to construct cross-batch similar-cell connected graphs using MNNs. (**a**) Closed-loop connection. (**b**) Transmitting connection. (**c**) Weak transmitting connection.

Closed-loop connection (Figure 11a): A1, A2, and A3 can perfectly form a connected graph if A1 in batch 1, A2 in batch 2, and A3 in batch 3 are all MNN pairs with each other.

Transmitting connection (Figure 11b): We found that similar cells across batch are transmittable (e.g., if A1 in batch 1 and A2 in batch 2 are MNNs, and A2 in batch 2 and A3 in batch 3 are MNNs, then A1 and A3 are similar cells). According to the transmissibility, A1, A2, and A3 can also form a connected graph.

Weak transmitting connection (Figure 11c): To make the connected graphs contain as many cells from different batches as possible, we loosened the transmissibility condition. The k-nearest neighbor algorithm is first executed within batches, and k within-batch neighbors are identified for each cell. If A in batch 1 and B in batch 2 are MNNs, A’ in batch 1 and C in batch 3 are MNN pairs, and A and A’ are k-nearest neighbors within batches, then B is a similar cell to C, and thus A/A’, B, and C can construct a connectivity graph.

By performing these three connection methods to construct connected graphs, the similarity of relationships of cells in different batches are obtained.

### 4.3. Correcting Batch Effects by GAN

Instead of selecting a batch as the “anchor” and transforming other batches to the “anchor” batch in turn as other MNN-based algorithms do, IMGG adopts the strategy of finding an intermediate batch and transforming all batches to the intermediate batch at the same time.

Firstly, we mapped the connected graphs obtained from the embedding space to the HVGs’ space, and randomly sampled each batch of cells on each connected graph to obtain a cohort of similar cells from different batches. To build the intermediate batch we designed three patterns, i.e., Mean, Max, and Min. For the Mean pattern, IMGG selects the mean value of each gene expression in the similar cell cohort as the synthesis target, which has the advantage of equalizing the differences in gene expression between batches and does not cause the corrected data to deviate from the normal range of values due to abnormalities in one batch. For the Max pattern, IMGG selects the maximum value of each gene expression in the similar cell cohort as the synthesis target, which has the advantage of combining the benefit points of different techniques when the batches are from different techniques and reflects a more comprehensive gene expression. For the Min pattern, IMGG selects the minimum value of each gene expression in the similar cell cohort as the synthesis target, which does not seem to be beneficial, but we did not remove it for the sake of algorithmic integrity. Using the synthesis point of each cohort as the target domain and the respective gene expression of the cells in the cohort as the source domain, a network was trained using GAN to transform from the source domain to the target domain, whereas the batch effects could be corrected later using the trained generator.

### 4.4. Model Details

**Figure 12:**
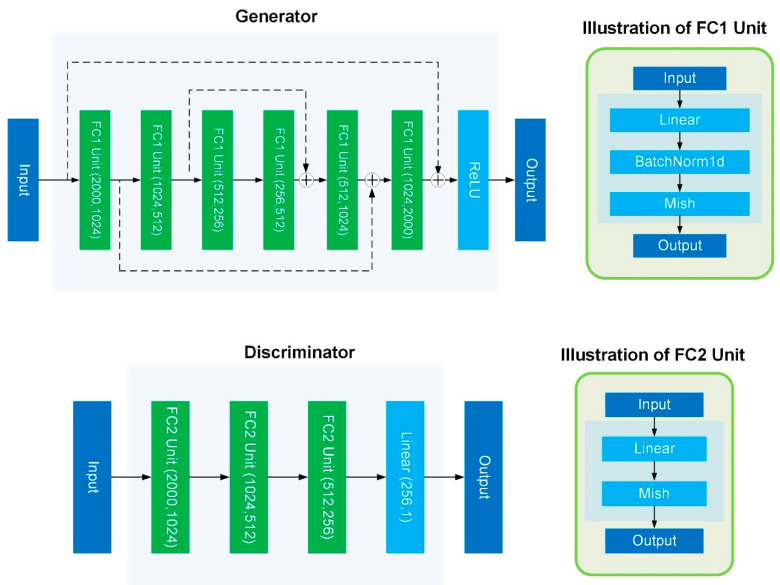
The network structure of GAN.

As shown in Figure 12, we used a residual fully connected layer as the generator of the GAN; each fully connected unit contains Linear, BatchNormal, and Mish activations [26]. Finally, the ReLU activation function was used to ensure that the output conformed to the gene expression distribution.

To facilitate and stabilize the GAN training process, adversarial losses were optimized via the WGAN-GP [27].

The loss function of the discriminator is:

(1) $$L_{adv}=\underset{\tilde{x}\sim {\mathbb{P}}_{g}}{E}\left[D\left(\tilde{x}\right)\right]-\underset{x\sim {\mathbb{P}}_{r}}{E}\left[D\left(x\right)\right]+\lambda \underset{\tilde{x}\sim {\mathbb{P}}_{\tilde{x}}}{E}\left[{\left({\left\|\nabla_{\tilde{x}}D\left(\tilde{x}\right)\right\|}_{2}-1\right)}^{2}\right]$$

The loss function of the generator is:

(2) $$L_{g}=-\underset{\tilde{x}\sim {\mathbb{P}}_{g}}{E}\left[D\left(\tilde{x}\right)\right]$$

However, in practice, we found that it would be difficult to integrate multiple batches of distributions using only the WGAN-GP loss, so we added a reconstruction loss to help GAN fit multiple batches of distributions better and faster, with good experimental results (Appendix A.4).

(3) $$L_{rec}=\underset{\tilde{x}\sim {\mathbb{P}}_{g},x\sim {\mathbb{P}}_{r}}{E}\left[{\left\|\tilde{x}-x\right\|}_{2}\right]\cdot n$$

where n is the number of genes.

We adopted the Adam optimizer [28] to train the networks, with a learning rate of 0.0002. The total time cost depends on the time spent building intermediate data and network-optimization parameters (epoch, batch size), and users can adjust all hyper-parameters to achieve better results.

All jobs are run on a Linux server configured with an Intel(R) Xeon(R) Gold 6226R CPU @ 2.90 GHz, 376 G DDR4 RAM, and a 32 G Tesla V100S GPU.

## Figures and Tables

**Figure A1:**
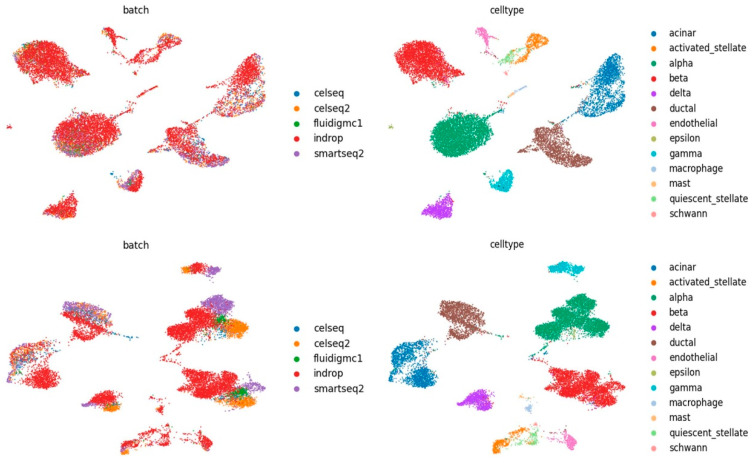
The first row plots the UMAP diagrams of IMGG-corrected data after adding reconstruction loss. The second row plots the UMAP diagrams of IMGG-corrected data without adding reconstruction loss.

**Figure A2:**
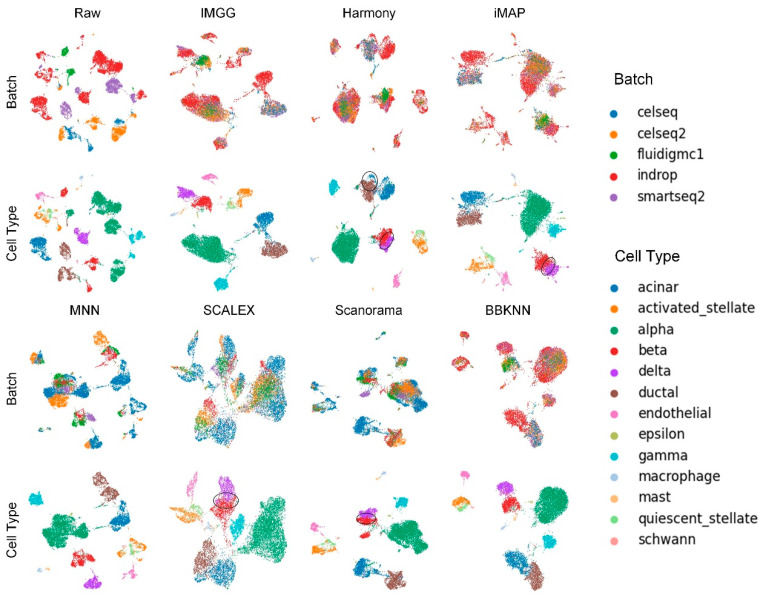
Qualitative evaluation of 7 batch-effect correction methods using UMAP visualization for Panc_rm dataset. The UMAP diagrams of raw data, IMGG, Harmony, and iMAP are plotted in the top half, and the UMAP diagrams of MNN, SCALEX, Scanorama, and BBKNN are plotted in the bottom half. Each half contains two rows of UMAP plots. In the first row, cells are colored by batch, and in the second by cell type.

**Figure A3:**
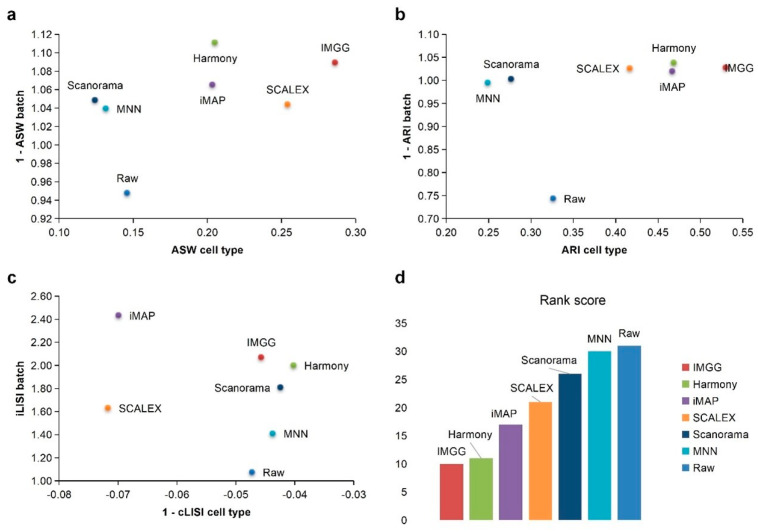
Quantitative evaluation of 6 batch-effect correction methods using the three-assessment metrics (**a**) ASW, (**b**) ARI, and (**c**) LISI on Panc_rm dataset. Methods appearing at the upper right quadrant of the ASW, ARI, and LISI plots are the good performing methods. (**d**) The sum of each method’s ranking on all evaluation metrics, with smaller values indicating better overall performance. Detailed data are recorded in Table A8.

## Data Availability

Not applicable.

## Appendix A

### Appendix A.1. Evaluation Indicators

To evaluate the effectiveness of batch correction, researchers have designed various evaluation metrics, such as ASW [14], ARI [15], and LISI [10], among others. Although each evaluation metric has its limitations, we believe that for the same data set, the algorithm is optimal only if it performs well on multiple evaluation metrics. The implementation details of each evaluation metric are as follows:

#### Appendix A.1.1. Average Silhouette Width (ASW)

The silhouette coefficient is a way of evaluating the effectiveness of clustering. Assuming that we have clustered the data by a certain algorithm, for one of the points, i, the silhouette coefficient is:

(A1) $$S\left(i\right)=\frac{b\left(i\right)-a\left(i\right)}{\max\left\{a\left(i\right),b\left(i\right)\right\}}$$

where a(i) is the average distance of other samples in the same category as it and b(i) is the average distance of samples in different categories that are closest to it. The value of the silhouette coefficient is between [−1, 1], the closer to 1 means that the cohesion and separation are relatively good. The average of the silhouette coefficients of all points is the total silhouette coefficient of the clustering result.

In our work, the ASW score was calculated in the python language environment using the “silhouette_score” function of the “sklearn.metrics” package and with the top 50 principal components of the corrected data as input. Using batch as the label, a lower ASW score indicates better batch mixing, and using cell type as the label, a higher ASW score indicates better cohesion of similar cells.

#### Appendix A.1.2. Adjusted Rand Index (ARI)

Given the actual category information C, assuming that K is the clustering result, a denotes the number of pairs of elements that are of the same category in both C and K, and b denotes the number of pairs of elements that are of different categories in both C and K, then the Rand index is

(A2) $$RI=\frac{a+b}{C_{2}^{n_{samples}}}$$

where C is the total number of element pairs that can be composed in the data set and RI takes values in the range [0, 1], with larger values implying that the clustering results match the true situation. For random results, RI does not guarantee that the score is close to zero. In order to achieve “the indicator should be close to zero in the case of randomly generated clustering results”, an adjusted Rand factor is proposed, which provides a higher degree of discrimination:

(A3) $$ARI=\frac{RI-E\left[RI\right]}{\max\left(RI\right)-E\left[RI\right]}$$

The ARI takes values in the range [−1, 1], with larger values meaning that the clustering results match the true situation.

In our work, K-Means (where k is the number of cell types) clustering was performed in the Python language environment using the “KMeans” function from the “sklearn.cluster” package and using the top 50 principal components of the corrected data as input. To assess cell-type purity using ARI, cell-type labels were compared to k-mean clustering results using the “adjusted_rand_score” function of the “sklearn.metrics” package, with high ARI scores corresponding to high cell-type purity. For batch-mixing assessment, only cells whose types were present in all batches were considered, and their respective batch labels were compared with the KMeans clustering labels, and a low ARI score indicated good mixing.

#### Appendix A.1.3. Local Inverse Simpson’s Index (LISI)

LISI is a metric for assessing the mix of batches and cell types. In the case of LISI integration (iLISI) to measure batch mixing, the index is computed for batch labels, and a score close to the expected number of batches denotes good mixing. The iLISI score is only computed for cells whose type appears in all batches. For cell-type LISI (cLISI), the index is computed for all cell-type labels, and a score close to 1 denotes that the clusters contain pure cell types. We calculated the iLISI and cLISI scores for each cell in the dataset and then determined the mean for comparison.

#### Appendix A.1.4. Differential Gene Expression Analysis (DEG)

To perform DEG analysis, we first preprocessed the data (including log2 normalization), then selected samples and grouped them, after which we calculated the difference in expression levels of target genes between different groups of samples (i.e., fold change). We used the stats function of the SciPy package in the Python language environment to perform t-tests to calculate the significance (*p*-value) of gene expression differences between samples. In our experiments, the threshold value of fold change was 1 and *p* was 0.05.

#### Appendix A.1.5. Uniform Manifold Approximation and Projection (UMAP) Visualization

We ran UMAP with the default number of neighbors using the Scanpy package in the Python environment to visualize the raw data and batch-corrected output.

### Appendix A.2. Datasets

**Table A1:** The real datasets used in this study.

Dataset	Description	Batch (Number of Cells)	Number of Cell Types	Genes	Overlap
PBMC	human peripheral blood mononuclear cells	pbmc_3p (8098)	12	33,694	True
		pbmc_5p (7378)	12		
Pancreas	human pancreas	Indrop (8569)	13	34,363	True
		smartseq2 (2394)	13		
		celseq2 (2285)	13		
		Celseq (1004)	13		
		fluidigmc1 (638)	13		
DC	human dendritic cells	Batch1 (283)	3	26,593	False
		Batch2 (286)	3		
Panc_rm	Panc_rm	Indrop (5147)	11	34,363	False
		smartseq2 (1898)	11		
		celseq2 (1808)	11		
		Celseq (725)	11		
		fluidigmc1 (592)	11		
PBMC_rm	PBMC_rm	pbmc_3p (150)	2	33,694	True
		pbmc_5p (150)	2		

We also generated simulated data using the splatter package in R environment [29], with “batch.facLoc” set to 0.1 and “batch.facScale” set to 0.15.

**Table A2:** The simulated datasets used in this study.

Number of Cells	Batch1:Batch2:Batch3:Bath4	Group1:Group2:Group3:Group4	Genes
1000	1:1:1:1	1:1:1:1	10,000
2000	1:1:1:1	1:1:1:1	10,000
5000	1:1:1:1	1:1:1:1	10,000
10,000	1:1:1:1	1:1:1:1	10,000
20,000	1:1:1:1	1:1:1:1	10,000
50,000	1:1:1:1	1:1:1:1	10,000
80,000	1:1:1:1	1:1:1:1	10,000
100,000	1:1:1:1	1:1:1:1	10,000

All the datasets that we used are available at https://github.com/dongzuoyk/IMGG (accessed on 14 January 2022).

### Appendix A.3. Comparison Methods

**Table A3:** The comparison methods used in this study.

Tools	Output	Language	Availability
iMAP [7]	Normalized gene expression matrix	Python	https://github.com/Svvord/iMAP (last access date: 12 February 2022)
MNN [6]	Normalized gene expression matrix	Python/R	https://github.com/MarioniLab/MNN2017 (last access date: 12 February 2022)
Scanorama [9]	Normalized dimension reduction vectors	Python/R	https://github.com/brianhie/scanorama (last access date: 12 February 2022)
Harmony [10]	Normalized feature reduction vectors	Python/R	https://github.com/immunogenomics/harmony (last access date: 12 February 2022)
SCALEX [8]	Normalized feature reduction vectors and Normalized gene expression matrix	Python	https://github.com/jsxlei/SCALEX (last access date: 12 February 2022)
BBKNN [12]	Connectivity graph and normalized dimension reduction vectors	Python	https://github.com/Teichlab/bbknn (last access date: 12 February 2022)

### Appendix A.5. Experiment 2

The Panc_rm dataset was composed by removing some cells in each of the five batches of the Pancrease dataset. We removed ductal and beta cells in ‘indrop’ batch, acinar and beta cells in ‘smartseq2′ batch, acinar and delta cells in ‘celseq’ batch, acinar and delta cells in ‘celseq’ batch, and acinar and delta cells in ‘fluidigmc1′ batch, thus constructing a five-batch non-overlapping dataset.

We used the Panc_rm dataset for our experiments and the UMAP visualization plots (Figure A2) showed that MNN performed the worst in terms of batch mixing, and there were “kissing effects” between beta and delta cells in SCALEX, Harmony, iMAP, and Scanorama. Acinar and ductal cells showed varying degrees of “kissing effects” in all methods, which may be attributed to the similarity of gene expression.

For ASW (Figure A3a), IMGG was second only to Harmony in terms of batch mixing and ahead of all other algorithms in terms of cell-type purity. For ARI (Figure A3b), all algorithms performed well in batch mixing (1 − ARI batch > 0.99), and IMGG scored highest in cell-type purity assessment. For LISI (Figure A3c), IMGG was second only to iMAP in batch-mixing assessment, and it took fourth place in cell-type purity, but the score difference with the top three was less than 0.01. Finally, based on the sum of the rankings of the assessment metrics (for fairness, if the score difference was less than 0.01, the ranking was considered the same), IMGG was ranked first (Figure A3d).

### Appendix A.6. Experiment 3

We performed differential expression analysis of B cells and CD4 T cells in the ‘pbmc_3p’ batch, the ‘pbmc_5p’ batch, and the IMGG-corrected data from the PBMC dataset, and obtained 36, 44, and 39 differentially expressed genes, respectively. We used the expression of these differentially expressed genes for ASW assessment on the raw data, the ‘pbmc_3p’ batch, the ‘pbmc_5p’ batch, and the IMGG-corrected data, respectively. The higher value of ASW indicates that the two cell types are more dissimilar. As can be seen from Table A4, the differentially expressed genes obtained using IMGG-corrected data had the highest ASW scores, which indicates that the IMGG-corrected data can be analyzed for differential expression and that the differentially expressed genes found are more reflective of cellular differences.

**Table A4:** ASW assessment of B cells and CD4 T cells using expression of differentially expressed genes.

Batch	$ASW_{DEG\_3p\_36}$	$ASW_{DEG\_5p\_44}$	$ASW_{DEG\_IMGG\_39}$
Raw	0.511844	0.505531	0.518933
pbmc_3p	0.496737	0.498288	0.511863
pbmc_5p	0.578334	0.574564	0.590644
IMGG-corrected	0.703968	0.70964	0.714085

DEG_3p_36: 36 differentially expressed genes obtained using the ‘pbmc_3p’ batch; DEG_5p_44: 44 differentially expressed genes obtained using the ‘pbmc_3p’ batch; DEG_IMGG_39: 39 differentially expressed genes obtained using the IMGG-corrected data.

To demonstrate the robustness of IMGG in terms of DEG, we performed the same experiments on NK and DC cells, as well as CD8 T cells and monocyte-CD14 cells in the PBMC dataset, and the Venn diagram (Figure A4) demonstrates that the differentially expressed genes obtained using IMGG-corrected data combine two batch characteristics.

### Appendix A.7. Detailed Evaluation Index Score Data

All assessment index scores were averaged five times and retained two decimal places.

**Table A5:** Details of quantitative assessment metric scores on the PBMC dataset.

Method	ASW|Rank		ARI|Rank		LISI|Rank		Total Ranking Score
	$1-ASW_{batch}$	$ASW_{celltype}$	$1-ARI_{batch}$	$ARI_{celltype}$	iLISI	1−cLISI	
Raw	0.93|2	0.15|5	0.99|1	0.52|6	1.02|7	−0.17|3	24
IMGG	0.99|1	0.23|2	0.99|1	0.60|3	1.71|1	−0.12|1	9
Harmony	0.99|1	0.19|3	0.99|1	0.60|3	1.68|2	−0.17|3	13
iMAP	0.99|1	0.17|4	0.99|1	0.56|5	1.51|4	−0.22|5	20
MNN	0.99|1	0.19|3	0.99|1	0.61|2	1.18|6	−0.17|3	16
SCALEX	0.99|1	0.26|1	0.99|1	0.57|4	1.57|3	−0.13|2	12
Scanorama	0.99|1	0.15|5	0.99|1	0.64|1	1.50|5	−0.21|4	17

**Table A6:** Details of quantitative assessment metric scores on the Pancreas dataset.

Method	ASW|Rank		ARI|Rank		LISI|Rank		Total Ranking Score
	$1-ASW_{batch}$	$ASW_{celltype}$	$1-ARI_{batch}$	$ARI_{celltype}$	iLISI	1−cLISI	
Raw	1.01|7	0.17|7	0.99|2	0.54|7	1.06|7	−0.05|1	31
IMGG	1.15|1	0.42|1	0.99|2	0.89|1	2.38|1	−0.05|1	7
Harmony	1.14|2	0.26|3	0.99|2	0.74|3	2.00|3	−0.05|1	14
iMAP	1.06|5	0.21|4	1.00|1	0.77|2	2.16|2	−0.07|3	17
MNN	1.11|3	0.18|6	0.99|2	0.55|6	1.38|6	−0.05|1	24
SCALEX	1.04|6	0.29|2	0.99|2	0.59|5	1.67|5	−0.06|2	22
Scanorama	1.09|4	0.19|5	0.99|2	0.64|4	1.85|4	−0.05|1	20

**Table A7:** Details of quantitative assessment metric scores on the DC dataset.

Method	ASW|Rank		ARI|Rank		LISI|Rank		Total Ranking Score
	$1-ASW_{batch}$	$ASW_{celltype}$	$1-ARI_{batch}$	$ARI_{celltype}$	iLISI	1−cLISI	
Raw	0.99|2	0.50|4	1.00|1	0.98|1	1.64|5	−0.02|1	14
IMGG	1.00|1	0.56|2	1.00|1	0.98|1	1.77|2	−0.02|1	8
Harmony	1.00|1	0.51|3	1.00|1	0.98|1	1.85|1	−0.02|1	8
iMAP	1.00|1	0.48|5	1.00|1	0.91|4	1.72|4	−0.04|3	18
MNN	0.99|2	0.50|4	1.00|1	0.96|2	1.63|6	−0.03|2	17
SCALEX	0.99|2	0.63|1	1.00|1	0.90|5	1.74|3	−0.03|2	14
Scanorama	1.00|1	0.44|6	1.00|1	0.92|3	1.55|7	−0.02|1	19

**Table A8:** Details of quantitative assessment metric scores on the Panc_rm dataset.

Method	ASW|Rank		ARI|Rank		LISI|Rank		Total Ranking Score
	$1-ASW_{batch}$	$ASW_{celltype}$	$1-ARI_{batch}$	$ARI_{celltype}$	iLISI	1−cLISI	
Raw	0.95|6	0.15|5	0.74|6	0.33|5	1.07|7	−0.05|2	31
IMGG	1.09|2	0.29|1	1.03|2	0.53|1	2.07|2	−0.05|2	10
Harmony	1.11|1	0.21|3	1.04|1	0.47|2	2.00|3	−0.04|1	11
iMAP	1.06|3	0.20|4	1.02|3	0.47|3	2.43|1	−0.07|3	17
MNN	1.04|5	0.13|6	0.99|5	0.25|7	1.41|6	−0.04|1	30
SCALEX	1.04|5	0.25|2	1.03|2	0.42|4	1.63|5	−0.07|3	21
Scanorama	1.05|4	0.12|7	1.00|4	0.28|6	1.81|4	−0.04|1	26
